# Lack of timely accrual information in oncology clinical trials: a cross-sectional analysis

**DOI:** 10.1186/1745-6215-15-92

**Published:** 2014-03-25

**Authors:** Aaron P Mitchell, Bradford R Hirsch, Amy P Abernethy

**Affiliations:** 1 Duke Cancer Institute & Center for Learning Health Care, Duke Clinical Research Institute 2400 Pratt St, Durham, NC 27705, USA; 2 Department of Internal Medicine, Duke University Hospital, 2301 Erwin Road, Rm 8254DN, Durham, NC 27710, USA; 3 Duke University Medical Center, Box 3436, Durham, NC 27710, USA

**Keywords:** Accrual, Cancer, Clinical trials, ClinicalTrials.gov, Renal cell carcinoma

## Abstract

**Background:**

Poor accrual is a significant barrier to the successful completion of oncology clinical trials; half of all phase 3 oncology trials close due to insufficient accrual. Timely access to accrual data fosters an understanding of successful trial design and can be used to inform the design of new clinical trials prospectively. Accrual statistics are available within research networks, such as the cancer cooperative groups, but comprehensive data reflecting the overall portfolio of cancer clinical trials are lacking. As a demonstration case, the purpose of this study was to quantify the public availability of accrual data across all recent renal cell carcinoma (RCC) trials.

**Methods:**

The database for the Aggregate Analysis of ClinicalTrials.gov (AACT) summarizes all trials registered between October 2007 and September 2010. In total, 108 trials of pharmacologic therapy for RCC were included. Accrual data on these trials were gathered via ClinicalTrials.gov (CTG), a manual review of resulting publications, and online surveys sent to principle investigators or trial coordinators.

**Results:**

In total, 26% (20 of 76) of trials listing a government, academic, or cooperative group (GAC) sponsor responded to the survey vs 0% (0 of 32) of those listing only industry sponsors. Across all methods, accrual data were available for only 40% (43 of 108) of trials, including 37% (28 of 76) of GAC trials and 47% (15 of 32) of industry trials. Moreover, 87% (66 of 76) of GAC trials were ongoing (open, actively recruiting, or of unknown status) vs 75% (24 of 32) of industry trials, while 9% (10 of 108) of trials were terminated or suspended.

**Conclusions:**

Despite extensive efforts (surveys, phone calls, CTG abstraction, publication searches), accurate accrual data remained inaccessible for 60% of the RCC trial cohort. While CTG reports trial results, ongoing accrual data are also critically needed. Poor access to accrual data will continue to limit attempts to develop a national summary of clinical trials metrics and to optimize the cancer clinical research portfolio.

## Background

We must improve accrual of study participants to clinical trials. Successful accrual ensures the appropriate use of limited research resources by enabling study completion. It also indicates the perceived value of a trial’s clinical questions and methodologies; efficient accrual signals that the results of a study are likely to be important and impactful.

Within oncology, poor accrual is a leading barrier to progress in clinical research [1]. Half of all phase 3 oncology trials close because of insufficient accrual [2], with only 2% of cancer patients participating [3,4]. Surveys and observational studies have identified trial characteristics that may predict accrual success, including cancer type, number of inclusion criteria, use of a placebo arm, randomization strategy, proximity to an academic center, and a managed care environment [2,5-8]. However, a detailed model of accrual success is lacking.

It is difficult to identify ways to align research priorities, trial methodologies, and recruitment networks to optimize accrual until, through a study of past experiences, we identify practices that positively affect accrual rates. Necessarily, the effective study of past clinical research requires that clinical trial results, most importantly data regarding recruitment rates and targets, be made publicly available in a timely fashion.

Monitoring of, and reaction to, past and ongoing accrual patterns enables the prospective improvement of accrual rates. At the institutional level, others have found that close monitoring of accrual, ‘aid[s] in continuously tracking and troubleshooting clinical trial accrual’ and have called for ‘a continuous feedback loop of information for sustaining the pipeline of clinical trials [9]’. The ability to track accrual on a larger scale across all clinical trials may yield similar improvements.

The systems in place for clinical trial reporting might be inadequate to facilitate the needed level of data transparency and availability. Currently, data are available only in pockets; for example, accrual to federally funded cancer cooperative group trials can be characterized, but these data are not publicly available and are difficult to interpret without access to trials run by different sponsors. In response to this problem, the development of registries, such as ClinicalTrials.gov (CTG), has been mandated by the US Food and Drug Administration (FDA) to capture data on the clinical research portfolio; the mandate acknowledges the importance of developing comprehensive data repositories that can be leveraged in order to maximize our societal investment in clinical research.

ClinicalTrials.gov requires registration of all phase 2 to 4 interventional drug or device trials that are conducted (in whole or in part) in the United States or are conducted under an investigational new drug application or investigational device exemption. Since its expansion under the FDA Amendments Act of 2007, Section 801, results reporting has also been mandatory. Required results include, at minimum, (1) adverse events, (2) outcome measures, (3) participant baseline characteristics, and (4) participant flow, which describes the number of subjects enrolling in and completing the trial. To comply with the law, results must be finalized within 1 year of the end of data collection [10].

These data, if updated and aggregated in a timely fashion, could be used to identify trial factors and strategies that produce successful achievement of recruitment goals. For example, prior studies have shown that streamlining the trial design process would increase successful accrual [1], and that development of a model for predicting accrual success would enable the early identification of trials unlikely to achieve sufficient accrual, allowing for trial redesign and saving scarce research resources [2]. For CTG to succeed as a public repository, facilitating research transparency, and for it to be useful in the secondary analysis of clinical trials, it is necessary that data reported on CTG be accurate, complete, and up-to-date.

The purpose of our research effort was to determine whether publicly available clinical trial data are sufficient to develop a comprehensive understanding of accrual. We tested the hypothesis that incomplete and delayed reporting of clinical trials would result in low availability of accrual data through public channels. As a proof of concept, we selected a manageable cohort of trials for thorough analysis. Renal cell carcinoma (RCC) was chosen, owing to the rapid evolution of treatments in the field and the number of trials supporting novel agents (seven new drugs from 2005 through 2012) [11].

## Methods

The Clinical Trials Transformation Initiative, a public-private partnership between Duke University and the FDA, recently created the AACT (Aggregate Analysis of ClinicalTrials.gov) database, a searchable database of trials registered in ClinicalTrials.gov (CTG) and intended to facilitate analysis of the clinical trials portfolio [12-14]. Detailed methods describing the creation of the AACT, including the oncology specific dataset, have been reported previously [12]. The final oncology dataset included 8,942 trials registered on CTG between 2007 and 2010. Analysis of the AACT database identified 108 trials focused on pharmacologic therapy for RCC.

We used CTG as the initial data source, since accrual data are supposed to be available there by public mandate. All clinical trials registered on CTG initially include an anticipated accrual goal, denoted as ‘estimated enrollment’. For either ongoing or completed trials, this figure may be updated to reflect the actual number of trial subjects accrued, denoted ‘enrollment’. Those trials that presented ‘enrollment’ rather than ‘estimated enrollment’ figures on CTG were counted as having reported accrual data. Additionally, the dates of the end of data collection, denoted ‘primary completion date’, and trial status (pre-enrollment, completed, terminated, and so on) are investigator-reported figures listed for each trial on CTG. These data were abstracted in June 2012.

Owing to low rates of reporting on CTG, additional efforts were made to extract accrual data from other publicly available sources. This led to the implementation of a supplemental structured survey of clinical trial teams and a review of resulting publications for accrual data (see Additional file 1). Approval from the institutional review board was obtained before beginning the survey. For the majority of trials registered on CTG, one or more persons are listed as the principal investigators or trial coordinators; email addresses for each of these persons were obtained either directly from CTG or from institutional websites or other publications authored by the same persons. Other trials did not list investigators but instead listed a ‘contact person’; in such cases, our survey was sent to these persons. At least one functional email address (no ‘bounce-back’ message when the survey was sent) was ultimately available for all but five trials; for four of these trials, the relevant parties were contacted using phone numbers listed on CTG, and for one trial, no contact information was available.

In March of 2012, a survey was sent via email to the contacts, as described, requesting information about accrual rates, trial sponsor, and completion status. For each trial, the survey was sent to each available email address, whether this was only one or more than one. For no trial did we receive more than one survey response. If the survey was not completed within two weeks, a reminder email was sent and then an attempt at telephone contact was made.

PubMed and Google Scholar were used to search for resulting publications. For each trial, separate searches, including (1) the ClincalTrials.gov identifier number, (2) the study title as listed on CTG, and (3) the names of persons listed as principle investigators or trial coordinators, were conducted. Resulting abstracts or papers were searched for reported accrual figures. The date of the last abstraction was 23 June 23 2012.

## Results and discussion

Abstraction from CTG yielded accrual data for 8% (6 of 76) of trials listing a government, academic, or cooperative group sponsor (GAC trials) and 47% (15 of 32) of trials listing an industry sponsor. The survey response rate was 26% (20 of 76) for GAC and 0% (0 of 32) for industry trials. The PubMed review provided additional data from 3% (2 of 76) of GAC and 0% (0 of 32) of industry trials. Overall, accrual data were obtained for only 40% (43 of 108) of trials, including 37% (28 of 76) of GAC and 47% (15 of 32) of industry trials (Table 1).

**Table 1:** Availability of updated accrual data by source (left) and clinical trial status as listed on ClinicalTrials.gov

**Source of accrual information**						**Trial status per CTG**				
**Primary sponsor**	**Total trials**	**Survey**	**ClinicalTrials.gov**	**Publication**	**Not available**	**Completed**	**Recruiting**	**Open; ongoing or not recruiting**	**Unknown**	**Terminated or suspended**
GAC	76	20 (26)	6 (8)	2 (3)	48 (63)	3 (4)	46 (61)	11 (14)	9 (12)	7 (9)
Industry	32	0 (0)	15 (47)	0 (0)	17 (53)	5 (16)	9 (28)	15 (47)	0 (0)	3 (9)
**Total**	**108**	**20 (19)**	**21 (19)**	**2 (2)**	**65 (60)**	**8 (7)**	**55 (51)**	**26 (24)**	**9 (8)**	**10 (9)**

Percentages shown in parentheses. Government, Academic, or Cooperative group.

According to CTG records, 87% (66 of 76) of GAC trials registered between 2007 and 2010 were still ongoing (open, actively recruiting, or of unknown status) at the time of abstraction in June 2012 vs 75% (24 of 32) of industry trials (Table 1). Across all sponsors, more trials were terminated or suspended (9%, 10 of 108) than were completed (7%, 8 of 108).

In total, 62% (67 of 108) of the trials had reached their primary completion date by the time we conducted data abstraction. Accrual data were available for 46% (29 of 67) of the trials past their primary completions dates, and for 34% (14 of 41) of the trials that had not yet reached primary completion, 25% (27 of 108) were more than one year past their primary completion date (Table 2). Of trials more than one year past primary completion, 32% (6 of 19) of GAC trials and 75% (6 of 8) of industry trials had reported accrual data on CTG.

**Table 2:** Accrual reporting on ClinicalTrials.gov by clinical trials more than 1 year past date of primary completion

**Primary sponsor**	**Total trials**	**Trials >1 year past primary completion**	**Final accrual data reported on ClinicalTrials.gov**	**Final accrual data not reported on ClinicalTrials.gov**
Government, academic, or cooperative group	76	19	6 (32)	13 (68)
Industry	32	8	6 (75)	2 (25)
**Total**	**108**	**27**	**12 (44)**	**15 (56)**

Percentages shown in parenthesis.

## Conclusions

Data presently available via CTG are inadequate, as they are often incomplete and difficult to obtain. In our cohort of RCC trials, only 19% (21 of 108) reported accrual information on CTG, while 56% (15 of 27) did not report accrual on CTG despite being more than 1 year past the date of primary completion, in violation of federal reporting requirements. After expanding our data acquisition to include surveys, phone calls, and publication searches, accrual data for RCC trials remained largely unavailable. The results of this study demonstrate that access to trial results remains a barrier to research on accrual patterns; even with the time and capability to search the web manually for published results and contact trial investigators by telephone individually, we were able to obtain accrual figures for only 40% of the cohort of RCC trials. Factors contributing to the low response rate included a reluctance to release this information to unknown parties and possible time constraints among research teams.

Participant accrual remains a challenge to clinical research. Without adequate data, we will struggle to improve the completion of trials, devise, and implement new strategies to enhance accrual, and monitor impact. Detailed clinical trial data need to be available, in order to support our societal investment in research. Furthermore, data should be available quickly, as a ‘real-time approach’ of adapting to accrual patterns may be optimal [9].

To facilitate trial completion, we must better understand drivers of accrual by developing systems to monitor ongoing accrual success. Accrual statistics are available within research networks, such as the cancer cooperative groups, but they are not comprehensive or publicly available. Some regionalized efforts have made impressive progress in systematically monitoring accrual [8], and have begun to identify systemic drivers of accrual rates and clinical trial success [7,15]. However, this work has been limited to trials under US National Cancer Institute sponsorship, and therefore does not describe the full breadth of the clinical trial infrastructure. For a comprehensive understanding, such research must have access to and include accurate data on all clinical trials.

ClinicalTrials.gov is well positioned to meet these needs, as one of its objectives is to facilitate standardized reporting of trial characteristics and results. It requires reporting of accrual figures; however, such results are not required until 1 year after data collection, precluding the study of ongoing trials [13]. Additionally, compliance with CTG reporting is poor, with only 10 to 22% of registered trials meeting mandatory requirements [16,17]. Compliance with results reporting is particularly low for phase II trials (10% compared with 32% of phase III trials) and publicly funded trials (8% compared with 40% of industry funded trials) [17]. Though we used a different metric for results reporting that focused only on accrual data, our comparable result of 40% reporting reaffirms an ongoing need to for improvement.

Although greater transparency in recruitment may allow for improvement in accrual over the long term, there may also be drawbacks. Early public availability of accrual figures for ongoing trials might lead to withdrawal of funding for those trials with slower-than-expected recruitment. While this might help to funnel resources towards trials more likely to produce meaningful results, it could also result in financial stress at the institutional level. It is also possible that patients might decide to enroll in trials based upon publicly reported accrual rates, potentially further depressing accrual in trials that are already accruing poorly; we 1anticipate that this is an unlikely scenario.

Efforts to increase the timeliness and completeness of reporting will help CTG meet its full potential as a central clearing house of clinical trial data, capable of supporting vital analysis of our research priorities and conduct. A requirement that clinical trial data be updated at predefined intervals would significantly increase the quality of data available in CTG. As clinical researchers, study sponsors, and a community at large, it is important that we share this information, recognizing its importance in advancing the conduct of clinical trials.

## Abbreviations

AACT: Aggregate Analysis of ClinicalTrials.gov; CTG: ClinicalTrials.gov; FDA: US Food and Drugs Administration; GAC: government, academic, or cooperative group; RCC: Renal cell carcinoma.

## Competing interests

The authors disclose the following conflicts of interest:

Aaron Mitchell – none;

Bradford Hirsch – research funding (Pfizer, Dendreon, Bristol-Meyers Squibb);

Amy Abernethy – compensated leadership role (Advoset, American Academy of Hospice and Palliative Medicine, Orange Leaf Associates), compensated advisory role (Novartis, Bristol-Myers Squibb, Pfizer), research funding (Alexion, Kanglaite, Biovex, DARA BioSciences, Mi-Co, Genentech, Helsinn Therapeutics, Lilly, Bristol-Myers Squibb, Amgen, Pfizer);

This work was unfunded. No other party was involved in the design and conduct of the study, data analysis, or preparation or review of the manuscript.

## Authors’ contributions

APM conducted survey, data collection, and results analysis, and drafted the manuscript. BRH performed results analysis and drafted the manuscript. APA formulated the study and helped to draft the manuscript. All authors read and approved the final manuscript.

## Authors’ information

Dr. Abernethy had full access to all of the data in the study and takes responsibility for the integrity of the data and the accuracy of the data analysis.

## Supplementary Material

Additional file 1 Survey outline. Click here for file
