# Supplementary material for: Lack of timely accrual information in oncology clinical trials: a cross-sectional analysis
Source: Trials. 2014 Mar 25;15:92. doi: 10.1186/1745-6215-15-92 (PMC3987844; doi:10.1186/1745-6215-15-92)
Supplement: Additional file 1 — Survey outline. [file 1745-6215-15-92-S1.docx]

**Survey Outline**

1. Identifiers
   1. Name
   2. Position
   3. Trial Name
2. Trial Dates (month & year)
   1. Date of trial opening
   2. Date of first accrual
   3. Date of final accrual, or goal date if applicable
   4. Date of trial completion, or goal date if applicable
3. Recruitment Data
   1. Total recruitment goal
   2. Final recruitment attained, if trial ended
   3. Recruitment per quarter
4. Other Trial Data
   1. Financial sponsor(s) of trial
   2. Conducting trial (academic, CRO, etc)
5. Resulting Publications
